# Time Trends in Pediatric Hospitalizations for Varicella Infection Are Associated with Climatic Changes: A 22-Year Retrospective Study in a Tertiary Greek Referral Center

**DOI:** 10.1371/journal.pone.0052016

**Published:** 2012-12-28

**Authors:** Elena Critselis, Panagiotis T. Nastos, Kalliopi Theodoridou, Maria Theodoridou, Maria N. Tsolia, Christos Hadjichristodoulou, Vassiliki Papaevangelou

**Affiliations:** 1 Second University Department of Pediatrics, “P. & A. Kyriakou” Children's Hospital, National and Kapodistrian University of Athens School of Medicine, Athens, Greece; 2 Laboratory of Climatology and Atmospheric Environment, Department of Geology and Geoenvironment, National and Kapodistrian University of Athens, Athens, Greece; 3 First University Department of Pediatrics, “Aghia Sophia” Children's Hospital, National and Kapodistrian University of Athens School of Medicine, Athens, Greece; 4 Department of Hygiene and Epidemiology, University of Thessaly School of Medicine, Thessaly, Greece; University of Ottawa, Canada

## Abstract

**Background/Aims:**

The transmission rate of air-borne infectious diseases may vary secondary to climate conditions. The study assessed time trends in the seasonality of hospitalized varicella cases in a temperate region in relation to climatic parameters prior to the implementation of universal varicella immunization.

**Methods:**

A retrospective descriptive study was conducted among all pediatric and adolescent varicella patients (n = 2366) hospitalized at the “Aghia Sophia” Children's Hospital during 1982–2003 in Athens, Greece. Date of infection was computed based on hospital admission date. Seasonal and monthly trends in the epidemiology of varicella infection were assessed with time series analysis (ARIMA modeling procedure). The correlation between the frequency of varicella patients and the meteorological parameters was examined by the application of Generalized Linear Models with Gamma distribution.

**Results:**

During 1982–2003, the occurrence of hospitalized varicella cases increased during summer (p = 0.025) and decreased during autumn (p = 0.021), and particularly in September (p = 0.003). The frequency of hospitalized varicella cases was inversely associated with air temperature (p<0.001). In contrast, the occurrence of hospitalized varicella cases was positively associated with wind speed (p = 0.009).

**Conclusions:**

Pediatric hospitalizations for varicella infection rates have increased during summer and decreased during autumn in the examined temperate region. Time trends in hospitalized varicella cases are associated with climatic variables.

## Introduction

Chickenpox, caused by the varicella zoster virus (VZV), constitutes a prevalent disease among pediatric populations, particularly in temperate regions [1], [2]. Prior to the implementation of universal infant vaccination for varicella, more than 90% of children in temperate regions presented with varicella infection by mid-adolescence [3]–[6]. In Europe, the annual hospitalization rate attributed to varicella infection ranges between 12.9 to 28.0 per 100,000 children [1]. Although varicella is considered a mild disease, approximately 2–6% of varicella patients present with severe and potentially fatal complications, including respiratory and neurological complications, such as encephalitis and cerebellar ataxia [1], [3].

VZV is primarily transmitted by direct contact and secondarily by airborne transmission [7]. The epidemiology of varicella infection is noted to be remarkably different between temperate and tropical regions, with incidence rates being lower in the latter [8], [9]. Therefore, while varicella is a disease of preschool and school-aged children in temperate regions, in the tropics it often affects older age groups [1], [10]–[12]. Moreover, in tropical regions the seroprevalence rate of VZV is higher in urban versus rural populations [12]–[14]. It has been postulated that ambient temperature and humidity negatively affect VZV transmission [10]. Moreover, in temperate European countries, the seropositivity rate of VZV is lower in those Mediterranean countries with the hottest climates of the region [1], [15]. However, other factors such as population density, nursery attendance and socioeconomic development also may influence the epidemiology of VZV in areas where universal vaccination has not yet been implemented [16]. To date, the association between the epidemiological trends in varicella infection and climatic factors (temperature, rainfall, humidity) has mainly been studied in tropical regions [12], [17]–[19]. Nowadays, while environmental factors are modified in temperate regions secondary to the ozone phenomenon, the investigation of a possible association with epidemiologic changes in varicella incidence may be vital for designing related evidence-based public health prevention strategies [20].

The study objectives were to assess the time trends in the epidemiology of hospitalized varicella cases in a temperate Mediterranean country during the pre-licensure period for the varicella vaccine when climate changes were notable [20]. In addition, the study objectives were to evaluate the association between the frequency of hospitalized varicella cases and climatic variables in the aforementioned study site.

## Methods

### Study Design and Population

A retrospective descriptive study was conducted in all pediatric patients aged ≤14 years hospitalized for varicella infection at the public tertiary “Aghia Sophia” Children's Hospital in Athens, Greece between Jan. 1, 1982 – Dec. 31, 2003. The study site is located in the Athens metropolitan area and serves approximately 650,000 children aged 0–14 years [21]. The study hypothesis was that the diminishment over time in hospitalized varicella cases could be associated with changes in climatic conditions in the region. Data was retrieved from the hospital admission data log records, rather than individual patient files. As a result, access to personal details, such as immigrant status, number of siblings, and/or nursery attendance, was not granted. Moreover, no exclusion criteria with respect to either socio-demographic variables and/or severity of disease manifestation were applied. The research protocol was approved by the Hospital Ethics Committee. Since no personal data was recorded for the purposes of the study, written informed consent for study participation was not required.

### Data Collection

Hospital records were utilized to identify pediatric patients with varicella infection and demographic characteristics were recorded. In order to account for the incubation period and to consequently evaluate date of infection, the following assumptions were made: (1) varicella incubation period extends 10–14 days and admission occurs during the first four days of illness; and, (2) admission for cerebellar ataxia occurs one week later (8–11^th^ day of illness). Thus, the following computations were applied to approximate the date of infection: (1) patients without cerebellar ataxia were infected 2 weeks prior to hospital admission; and, (2) patients with cerebellar ataxia were infected 3 weeks prior to hospital admission. The derived estimated date of varicella infection was utilized for all analyses.

The climatic variables examined were air temperature (°C), relative humidity (%), and wind speed (m/s). Mean monthly values of meteorological data were extracted from historical daily records for the period 1982–2003 from the meteorological station of the National Observatory of Athens which is located on the Hill of Nymphs near the centre of Athens (λ = 23° 43′ E, φ = 37° 58′ N, h = 107 m a.m.s.l.).

### Statistical Analysis

Time series analyses (ARIMA modeling procedure) were applied to assess time trends in the occurrence of hospitalized varicella cases according to both season and month, respectively. The correlation between the frequency of hospitalized varicella cases and climatic parameters was examined by the application of Generalized Linear Models (GLM) with Gamma distribution [22]–[23]. Varicella cases fitted well to the Gamma distribution, after being checked by the application of the Kolmogorov-Smirnov test (statistic d). The modeling procedure used as a dependent variable the mean monthly number of hospitalized varicella patients, while climatic parameters were included separately as independent covariates. The goodness-of-fit of the models was evaluated through deviance residuals [22]. A p-value (*p*) of ≤0.05 was considered as the criterion for statistical significance. Analyses were conducted with SPSS version 17.0.

## Results

### Time trends in pediatric hospitalizations for varicella infection

During 1982–2003, 2366 pediatric patients were hospitalized with varicella infection in the temperate region examined. Overall, no significant change in the occurrence of hospitalized varicella cases was observed during the study period.

The occurrence of hospitalized varicella cases was highest during spring, exceeding two fifths of all cases, and winter (Table 1). However, no significant changes in the frequency of hospitalized cases were observed during either of the aforementioned seasons. In contrast, the proportion of hospitalized varicella cases increased during summer (p = 0.025) and decreased during autumn (p = 0.021). In particular, the mean increase in the proportion of hospitalized varicella cases during summer was 0.49% per year, while the mean decrease in the proportion of cases during autumn was 0.56% per year. Also, an inverse trend with regard to hospitalized varicella cases was observed during the autumn months (Table 1), as they were reduced to approximately half upon the conclusion of the study period. This trend was primarily attributed to the diminishment in varicella infection rates during the September months (Table 2).

**Table 1:** Seasonal time trends in pediatric hospitalized varicella cases in Athens, Greece (n = 2366).

Year	1982–83 (n = 96) n (%)	1984–85 (n = 153) n (%)	1986–87 (n = 245) n (%)	1988–89 (n = 267) n (%)	1990–91 (n = 243) n (%)	1992–93 (n = 195) n (%)	1994–95 (n = 211) n (%)	1996–97 (n = 218) n (%)	1998–99 (n = 216) n (%)	2000–01 (n = 233) n (%)	2002–03 (n = 289) n (%)	p-value
Spring	33 (33.4)	62 (40.5)	122 (49.8)	130 (48.7)	90 (37.0)	89 (45.6)	101 (47.9)	88 (40.4)	92 (42.6)	104 (44.6)	107(37.0)	0.949
Summer	11 (11.4)	26 (17.0)	34 (13.9)	52 (19.5)	38 (15.6)	42 (21.5)	37 (17.5)	35 (16.0)	38 (17.6)	71 (30.5)	61 (21.1)	***0.025***
Fall	21 (21.9)	26 (17.0)	16 (6.5)	19 (7.1)	22 (9.0)	20 (10.3)	19 (9.0)	29 (13.3)	16 (7.4)	10 (4.3)	31 (10.7)	***0.021***
Winter	31 (32.3)	39 (25.5)	73 (29.8)	66 (24.7)	93 (38.3)	44 (22.6)	54 (25.6)	66 (30.3)	70 (32.4)	48 (20.6)	90 (31.1)	0.961

**Table 2:** Monthly time trends in pediatric hospitalized varicella cases in Athens, Greece (n = 2366).

	1982–83 (n = 96) n (%)	1984–85 (n = 153) n (%)	1986–87 (n = 245) n (%)	1988–89 (n = 267) n (%)	1990–91 (n = 243) n (%)	1992–93 (n = 195) n (%)	1994–95 (n = 211) n (%)	1996–97 (n = 218) n (%)	1998–99 (n = 216) n (%)	2000–01 (n = 233) n (%)	2002–03 (n = 289) n (%)	p-value
Jan.	9 (9.4)	16 (10.4)	27 (11.0)	18 (6.7)	20 (8.2)	9 (4.6)	18 (8.5)	21 (9.6)	25 (11.6)	13 (5.6)	27 (9.3)	0.792
Feb.	8 (8.3)	10 (6.5)	29 (11.8)	27 (10.1)	23 (9.5)	20 (10.2)	25 (11.8)	20 (9.2)	28 (13.0)	18 (7.7)	42 (14.5)	0.476
Mar.	12 (12.5)	21 (13.7)	38 (15.5)	35 (13.1)	34 (14.0)	17 (8.7)	25 (11.8)	28 (12.8)	30 (13.9)	27 (11.6)	65 (22.5)	0.902
Apr.	10 (10.4)	23 (15.0)	43 (17.6)	37 (13.8)	21 (8.6)	26 (13.3)	34 (16.1)	24 (11.0)	24 (11.1)	30 (12.9)	28 (9.7)	0.933
May	11 (11.4)	18 (11.8)	41 (16.7)	58 (21.7)	35 (14.4)	46 (23.6)	42 (19.9)	36 (16.5)	38 (17.6)	47 (20.2)	14 (4.8)	0.799
Jun.	6 (6.2)	9 (5.9)	24 (9.8)	33 (12.4)	26 (10.7)	37 (19.0)	22 (10.4)	26 (11.9)	31 (14.4)	55 (23.6)	45 (15.6)	0.214
Jul.	4 (4.2)	11 (7.2)	9 (3.7)	12 (4.5)	8 (3.3)	4 (2.0)	12 (5.7)	5 (2.3)	7 (3.2)	15 (6.4)	14 (4.8)	0.482
Aug.	1 (1.0)	6 (3.9)	1 (0.4)	7 (2.6)	4 (1.6)	1 (0.5)	3 (1.4)	4 (1.8)	0 (0.0)	1 (0.4)	2 (0.7)	0.941
Sept.	4 (4.2)	6 (3.9)	3 (1.2)	5 (1.9)	7 (2.9)	1 (0.5)	1 (0.5)	4 (1.8)	3 (1.4)	2 (0.8)	6 (2.1)	***0.003***
Oct.	7 (7.3)	10 (6.5)	8 (3.3)	2 (0.7)	9 (3.7)	2 (1.0)	4 (1.9)	12 (5.5)	5 (2.3)	3 (1.3)	7 (2.4)	0.112
Nov.	10 (10.4)	10 (6.5)	5 (2.0)	12 (4.5)	6 (2.5)	17 (8.7)	14 (6.6)	13 (6.0)	8 (3.7)	5 (2.1)	18 (6.2)	0.063
Dec.	14 (14.6)	13 (8.5)	17 (6.9)	21 (7.9)	50 (20.6)	15 (7.7)	11 (5.2)	25 (11.5)	17 (7.9)	17 (7.3)	21 (7.3)	0.907

### Hospitalizations for varicella infection and climatic factors

**Figure 1:**
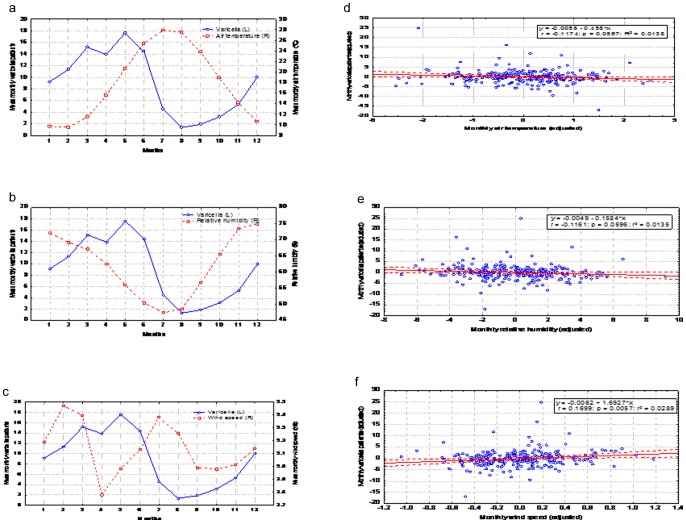
Association between climatic variables and hospitalization rates for varicella infection among children in a Greek tertiary care center (1982–2003). **a.** Intra annual variation of mean monthly air temperature and hospitalized varicella patients for the period 1982–2003. **b.** Intra annual variation of mean monthly relative humidity and hospitalized varicella patients for the period 1982–2003. **c.** Intra annual variation of mean monthly wind speed and hospitalized varicella patients for the period 1982–2003. **d.** Scatter plot between mean monthly varicella patients (adjusted for season and trend) and air temperature for the period 1982–2003. **e.** Scatter plot between mean monthly varicella patients (adjusted for season and trend) and relative humidity for the period 1982–2003. **f.** Scatter plot between mean monthly varicella patients (adjusted for season and trend) and wind speed for the period 1982–2003.

The overall intra-annual mean monthly variation of meteorological variables during the study period examined are presented in Figures 1a–1f. Mean monthly air temperature was inversely related with the frequency of hospitalized varicella cases (Figure 1a). However, when adjusted for seasonality and trend, mean air temperature was not significantly associated with the monthly frequency of hospitalized varicella patients (Figure 1d). Similar findings were observed for relative humidity (Figures 1b and Figure 1e). In contrast, mean monthly wind speed was positively correlated with the frequency in hospitalized varicella cases (Figure 1c), even following adjustment for seasonality (Figure 1f).

GLM with Gamma distribution (Kolmogorov-Smirnov statistical index d = 0.08) were applied using as the dependent variable the mean monthly number of hospitalized varicella patients and as independent covariates the individual climatic parameters. Thus, three GLM were constructed, each one including only one independent covariate, in order to examine the individual impact of each climatic covariate on varicella hospitalizations. The non-multicollinearity between the three independent variables was investigated by the application of the multicollinearity index VIF. The term of non-multicollinearity between the three independent variables T, RH and V was fully fulfilled (Table 3).

**Table 3:** Non-multicollinearity test results between the three independent variables: air temperature (T), relative humidity (RH) and wind speed (V).

Combination of the independent variables	Square of multiple correlation coefficient (R^2^)	VIF
T = f(RH, V)	0.166	1.20
RH = f(T, V)	0.173	1.21
V = f(T, RH)	0.219	1.28

The results of the GLM applied are presented in Table 4. A significant correlation between the mean monthly number of varicella hospitalised patients and the climatic parameters was established. Specifically, a decrease by an increment of 1°C in mean monthly air temperature was associated with a 3% increase in the number of hospitalized varicella cases. Furthermore, it is noteworthy that an increase by 1 m/s in mean monthly wind speed was associated with a 20% increase in the probability of hospitalization. On the other hand, no significant association was established between hospitalized varicella cases and relative humidity (Table 4).

**Table 4:** Generalized Linear Models (GLM) with Gamma distribution for assessing the association between mean monthly number of varicella patients and meteorological parameters.

Parameter	Estimate	Standard Error	p-value
Air temperature (^o^C)	−0.030	0.008	*<0.001*
Relative humidity (%)	−0.001	0.005	0.916
Wind speed (m/s)	0.181	0.070	*0.009*

## Discussion

The present study evaluated the time trends in pediatric hospitalizations for varicella infection in a temperate region prior to the implementation of universal VZV immunization among infants and during which period climate changes were notable [20]. The study also evaluated the association between the occurrence of hospitalized varicella cases and climatic variables during the aforementioned study period. The main study findings indicate that the occurrence of pediatric hospitalized varicella cases increased notably in the summer and decreased in the autumn seasons during the period 1982–2003. In particular, the most notable effects were observed in the September months of the period examined. The evidence provided supports that modifications in hospitalized varicella cases during the period 1982–2003 in the examined temperate Mediterranean region were associated with climatic factors. Specifically, the occurrence of hospitalized varicella cases was inversely associated with air temperature (p<0.001) and positively associated with wind speed (p = 0.009). No statistically significant relationship was revealed between hospitalized varicella cases and relative humidity.

Changes in the epidemiology of varicella infection, particularly in tropical regions, have been attributed to demographic changes, including increased population density and urbanization [2], [14]. Seroprevalence rates of varicella infection have been observed to be higher among urban populations, regardless of the population"s age distribution [8], [13]. It has been proposed that in urban tropical regions an elevated population density may partially overcome the transmission-interrupting effect of a tropical climate [12]–[13]. Specifically, urban settings may enable enhanced social interaction and population mobility, both within and between communities, and hence facilitate viral transmission [12]. Such evidence is corroborated by findings in temperate countries indicating that children with enhanced social interaction, such as that incurred by their attendance in nurseries, are more likely to contract varicella infection [15]. In the present reference population, a significant influx of immigrants was observed following the 1990′s. Immigrant children are more likely to be hospitalized for varicella complications [15]. However, as compared to Greek children, immigrant children have lower day care attendance rates and consequently contract varicella at an older age [15]. Since the mean age of hospitalized patients for varicella infection has not significantly changed during the study period [15], it is upheld that the aforementioned demographic changes did not markedly affect the epidemiological trends of hospitalizations for varicella infection in the population examined.

The study findings indicated that significant changes in the epidemiology of varicella infection have occurred during the seasons of summer and autumn. In particular, a diminishment in hospitalized varicella cases was observed during the September months. These findings corroborate with proposed hypotheses positing that changes in varicella infection rates in urban regions may be attributed to the combined effects of atmospheric air pollution and ultraviolet radiation [24].

The present study findings indicated that the modifications in the occurrence of hospitalized varicella cases in the evaluated temperate Mediterranean region were associated with climatic variables. In particular, the occurrence of hospitalized varicella cases was inversely associated with air temperature. Moreover, the frequency of hospitalized varicella cases was proximally associated with wind speed. Interestingly, although during the summer months of the period 1982–2003 an increase in mean air temperature was documented in the evaluated region, a concomitant increase in hospitalized varicella cases was also observed. However, the GLM analysis indicated that the potential effect of wind speed upon the occurrence of hospitalized varicella cases is substantially greater than that of mean air temperature. This is corroborated by the origin of the Greek term for “chickenpox” (“anemevlogia”) which is derived from the combination of the terms “anemos” ( = wind) and “evlogia” ( = small pox), indicating that the association between wind speed and varicella infection has been empirically suspected for several centuries. Therefore, it is upheld that the observed increase in hospitalized varicella cases in the summer months of the period 1982–2003 is more likely to be attributable to mean wind speed rather than mean air temperature.

Differential seroprevalence rates in varicella infection have been partly attributed to climatic factors, particularly in relation to temperature and humidity [10], [25]. Correlations between varicella incidence rates with both temperature and rainfall have been observed [2]. In tropical regions, evidence supports that the mean of extreme minimum temperatures is most proximally associated with increases in VZV seroprevalence. Due to the heat sensitivity of VZV, the virus may be inactivated at higher temperatures [12]. Hence, the viral transmission potential may be markedly reduced in countries with elevated mean temperatures and humidity [8], [14]. This has been primarily corroborated by the lower VZV seroprevalence rates observed in tropical regions [2].

Similarly, lower varicella seroprevalence rates in southern European countries have been attributed to the effects of the mild Mediterranean climate upon disease transmission potential [1]. It has been suggested that both the high levels of humidity and diminishment in the seasonal variations in temperature, particularly in countries proximal to the equator, may be associated with a uniform distribution of varicella cases throughout the year [8]. It is proposed that the observed epidemiological changes in the study region may be partly attributed to climatic changes which have resulted in the modifications of mean air temperature, relative humidity and, in particular, wind speed during the summer and autumn seasons [20]. However, mounting evidence exists supporting that the risk of infection by airborne viruses via aerosol transmission may be equivalent to that of direct transmission [26], [27], particularly in settings with low humidity [28]. The study findings indicated that the peak incidence rate remained greatest during the spring season throughout the time period evaluated. This contrasts findings in tropical regions which indicate that the peak incidence of varicella infection occurs during the cooler months of the year [12]. Thus, it may be inferred that although climatic changes in the study region have potentially influenced the seasonal distribution of varicella infection, the disease epidemiology still concurs with that of other temperate countries.

The strengths of present study include that it is the first of its kind to examine time trends in the epidemiology of pediatric hospitalized varicella cases over an extended time period in a temperate Mediterranean country. Data collected include more than four fifths of all patients hospitalized with varicella infection during the study period in the region [21]. In addition, the study included children hospitalized for varicella infection prior to the introduction of universal VZV vaccination in 2004. Hence, any changes observed in the epidemiological trends of varicella infection cannot be attributed to the introduction of the VZV universal vaccination. However, due to the retrospective nature of the study design applied, the etiological association between climatic changes and varicella infection cannot be conclusively established. Moreover, the potential confounding effects of socioeconomic factors upon the association of interest could also not be evaluated due to the retrospective study design applied. Finally, the present study cannot account for varicella infection cases contracted through direct cutaneous contact, rather than through the transmission via aerosolized droplets. Additional longitudinal studies are necessary to evaluate the interactive effects between demographic, climatic, and epidemiological changes, respectively, of other airborne viral diseases upon infection rates among children in temperate regions.

In conclusion, in a temperate Mediterranean region where significant variations in seasonality occurred during the pre-licensure period for the varicella vaccine (1982–2003), the occurrence of pediatric hospitalized varicella cases significantly increased during the summer and decreased during the autumn seasons. The observed epidemiological changes in the time trends of pediatric hospitalized varicella cases may be attributed to climatic changes in the region. It is hypothesized that time trends in the epidemiology of pediatric hospitalized cases for other infectious diseases which are similarly transmitted through indirect airborne transmission, such as influenza [29], may have also been affected by modifications in climatic variables [27], [28]. Therefore, public health interventions targeted at diminishing such infectious diseases among pediatric populations in the region should also account for the potential notable effects of modification of climatic variables upon the epidemiology of disease attributable hospitalizations [26].
